# Sipeimine reduces ethanol-induced gastric ulcer in mice by suppressing Jak-Stat activation and restoring gut microbiota balance

**DOI:** 10.1038/s41598-025-12050-2

**Published:** 2025-08-06

**Authors:** Xia Yang, Yue Li, Bing Bai, Qinlei Fan, Fei Liu, Shimin Xie, Yaru Li, Xiao Li, Jicheng Han, Yiquan Li, Guangze Zhu, Yilong Zhu, Ningyi Jin

**Affiliations:** 1 Department of Pathology and Pathophysiology, College of Medicine, Yanbian University, Yanji, 136200 Jilin China; 2 Key Laboratory of Jilin Province for Traditional Chinese Medicine Prevention and Treatment of Infectious Diseases, College of Integrative Medicine, Changchun University of Chinese Medicine, Changchun, 130117 PR China; 3 Jiangsu Co-innovation Center for Prevention and Control of Important Animal Infectious Diseases and Zoonoses, Yangzhou, 225009 China; 4 Animal Health and Epidemiology center, Qingdao, 266000 China

**Keywords:** Sipeimine, Gastric ulcer, Jak-stat pathway, Th17/Treg cell balance, Gut-gastro microbiota, Drug discovery, Plant sciences, Gastroenterology, Health care

## Abstract

Long-term excessive alcohol intake can directly injure the gastroduodenal mucosa, causing gastric erosions, gastric ulcers, and gastrorrhagia. Fritillaria ussuriensis Maxim is a famous traditional Chinese medicine and health food produced in China. Sipeimine is an alkaloidal component of Fritillaria ussuriensis Maxim. This research aimed to investigate the protective effects of sipeimine on ethanol-induced gastric ulcers in mice. The results displayed that sipeimine could alleviate gastric tissue damage and decrease the levels of SOD, MDA, IL-6, IFN-γ, TNF-α, and IL-1β. Sipeimine treatment also adjusted macrophage polarization and the balance of Th17/Treg cell by reducing the expression of Jak1/2, p-Jak1/2, Stat1/3, and p-Stat1/3. Moreover, sipeimine could increase the abundance of *Lactobacillus_johnsonii* and decrease the abundance of *Bacteroides_vulgatus* in the gut microbiota. Meanwhile, sipeimine treatment significantly decreased the abundance of *Rodentibacter_heylii* and *Streptococcus_cuniculi* in the gastric microbiota. In conclusion, sipeimine can improve gastric ulcers by suppressing the Jak-Stat pathway, reversing gut-gastro microbiota dysbiosis, inhibiting macrophage M1 polarization, maintaining the balance of Th17/Treg cell, and lessening sustained inflammatory injury.

**Supplementary Information:**

The online version contains supplementary material available at 10.1038/s41598-025-12050-2.

## Introduction

Drinking has become an essential part of interpersonal communication, which has led to an increase in the prevalence of alcohol-related diseases^1^. Long-term excessive alcohol intake can directly injure the gastroduodenal mucosa to cause gastric erosions, gastric ulcers, and gastrorrhagia^2^. However, the mechanism of alcohol-induced gastric mucosal injury has not been entirely illuminated. Numerous investigations have exposed that alcohol-related gastric mucosal injury is usually associated with the activation of pro-inflammatory reactions (such as Stat1-regulated macrophage M1 polarization^3,4^ and Stat3 affected Th17 cell differentiation^5,6^), which can intensify gastric mucosal inflammatory injury.

The primary strategies for treating gastric ulcers include restoring the injured gastric mucous membrane, controlling pathogenetic microorganisms, and reducing or neutralizing acid secretion^7^. The most commonly used strategy to treat gastric ulcers is quadruple therapy including bismuth, two types of antibiotics, and proton pump inhibitors^8^. However, the limitations of quadruple therapy (high relapse rate, side effects, and resistance of pathogenetic microorganism) have increased the demand for new therapies^9^. Modern medical research has shown that Chinese medicine treatment has the advantages of low toxicity and multi-channel and multi-targeting effects, which can be combined with quadruple therapy to coordinate the treatment of gastric ulcers^10,11^. Therefore, it is of great research significance to screen high efficiency and low toxicity monomers from Chinese medicine, which can increase therapeutic effectiveness and improve patients’ quality of life with gastric ulcers.

**Fig. 1:**
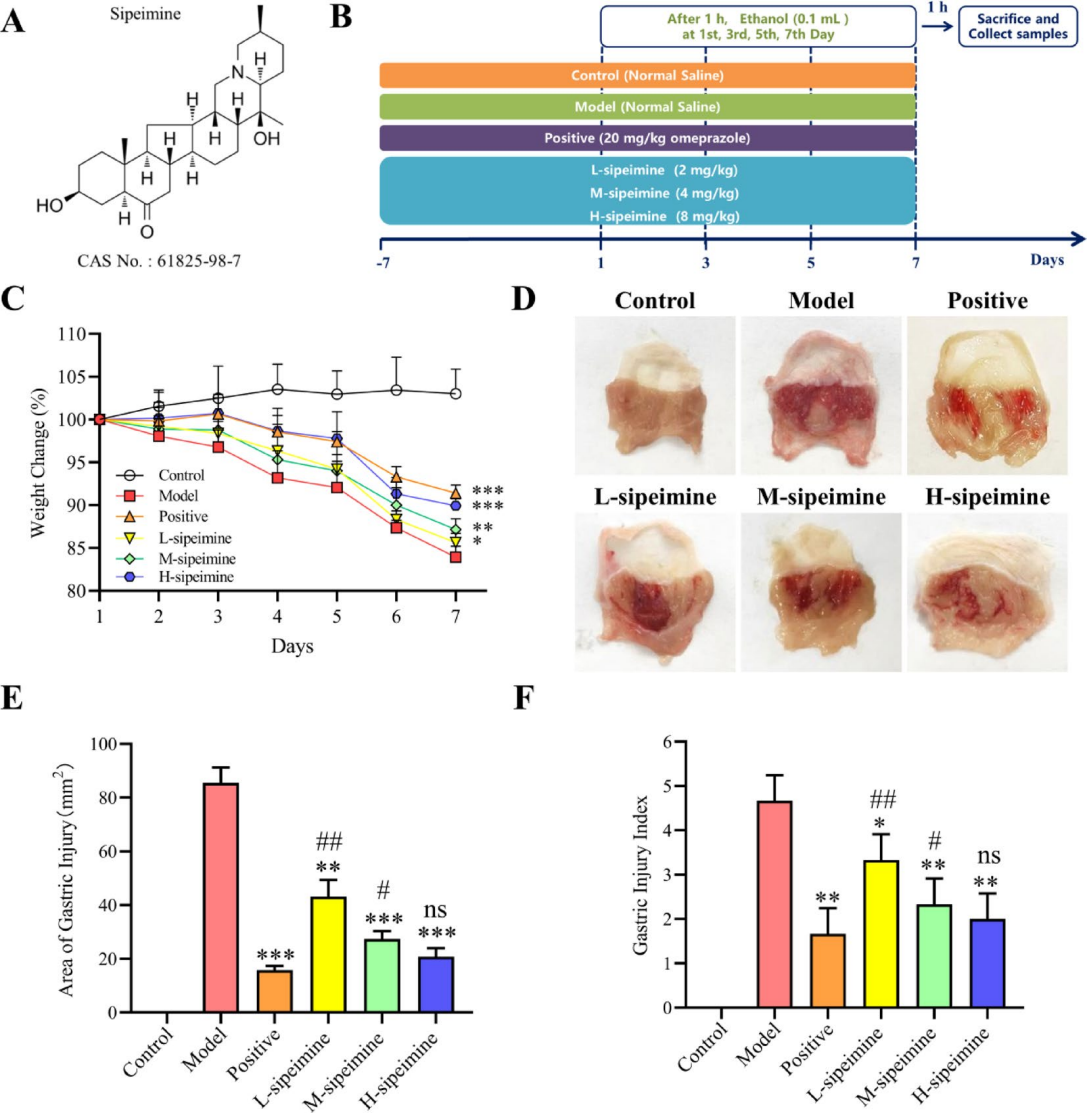
Effects of sipeimine on gastric ulcer mice. (**A**) Sipeimine chemical structures. (**B**) Experimental protocol. (**C**) The body weight changes. (**D**) Image of the stomach. (**E**) Area of gastric injury. (**F**) Gastric injury index. Data are presented as the mean ± standard deviation (SD; *p < 0.05, **p < 0.01, ***p < 0.001 compared to the model group; ^#^p< 0.05, ^##^p < 0.01 compared to the positive group; ns, not significant)

*Fritillaria ussuriensis Maxim* has been used extensively for over 2000 years, both in the food industry and as a medicinal plant. It has previously been observed that *Fritillaria ussuriensis Maxim* extracts have hypotensive effect^12^, antioxidant activity^13^ and immunological activity^14^. Sipeimine (Fig. 1A) is an alkaloid component in *Fritillaria ussuriensis Maxim* with various pharmacological properties, including anti-asthma^15^, anti-inflammation^16^, anti-tumor^17^, and anti-tussive effects^18^. In this study, based on the Jak-Stat pathway-regulated proinflammatory response and gut-gastro microbiota dysbiosis, we explored the effect and mechanism of sipeimine on ethanol-induced gastric ulcers in mice.

## Materials and methods

### Materials and reagents

Sipeimine (No. HY-N0696, purity ≥ 98.0%) was supplied by MCE (Shanghai, China) and dissolved as instructed by the manufacturer. Anhydrous ethanol was supplied by MERCK (Beijing, China). Jak1, Jak2, p-Jak1, p-Jak2, Stat1, Stat3, p-Stat1, p-Stat3, CD68, CD11b, FoxP3, and IL-17 A antibodies were supplied by Cell Signaling Technology (Shanghai, China). SOD, MDA, IL-6, IFN-γ, TNF-α, and IL-1β mouse ELISA kits were supplied by SINOBESTBIO (Shanghai, China). Tribromoethanol was purchased from Sigma-Aldrich (Shanghai, China).

### Animals and treatment

The current study is reported in accordance to ARRIVE guidelines. The experimental protocols were approved by the Institutional Animal Care and Use Committee of Changchun University of Chinese Medicine (No. 2023187). Weight Loss Threshold : euthanasia using carbon dioxide must be performed when mice exhibit, ≥20% loss of initial body weight and inability to maintain voluntary food/water intake.

C57BL/6J male mice, 8 weeks of age, were purchased from SiPeiFu Biotechnology (Beijing, China). All mice were randomized in 6 groups (*n* = 10): control (normal saline, i.g), model (0.1 mL anhydrous ethanol, i.g), positive (0.1 mL anhydrous ethanol + 20 mg/kg omeprazole, i.g), L-sipeimine (0.1 mL anhydrous ethanol + 2 mg/kg, low-dose, i.g), M-sipeimine (0.1 mL anhydrous ethanol + 4 mg/kg, middle-dose, i.g), and H-sipeimine (0.1 mL anhydrous ethanol + 8 mg/kg, high-dose, i.g).

Control group mouse was treated with the solvent every day, whereas the sipeimine groups treated with doses of sipeimine for 1 week. The experimental groups were subsequently treated intragastrically with 0.1 mL anhydrous ethanol at 2 h after administration every 2 days for 7 days (Fig. 1B). Mice were weighed at 8:30 AM every day for 7 days to monitor weight changes. Retro-orbital blood sampling was performed for ELISA analysis. After euthanized with carbon dioxide, the stomachs and cecal were excised, and their contents were collected for microbiome analysis. Use a vernier caliper to measure the major and minor axes of the gastric ulcer for irregular shapes, segmentally measure the main damaged area. The size and length of the linear gastric lesion or the gastric lesion itself were used to determine the degree of gastric mucosal injury. The area of gastric injury was determined in mm^2^, and the gastric mucosal injury index was computed as the sum of the gastric mucosal injury scores of each group/group number 100%^19^. Subsequently the stomach was divided into two portions for subsequent protein extraction (Western Blot) and histological analysis (HE/AB staining).

The samples (gastric tissues, serum, cecal, and gastric contents) were collected 6 h later on the last day of anhydrous ethanol stimulation after anesthetized with tribromoethanol and euthanized with carbon dioxide.

### HE and AB staining

The gastric tissues were fixed in a 4% paraformaldehyde solution for 24 h for 4℃, embedded in paraffin, sliced, dewaxed, and hydrated. HE or AB staining, dehydration, sealing, successive photographing, and histological evaluation of gastric tissue was performed as reported in the literature^19,20^.

### ELISA detection

Serum was collected from each group. The SOD, MDA, IL-6, IFN-γ, TNF-α, and IL-1β levels were detected by respective commercial ELISA kits following the manufacturer’s guidelines. The test serum was diluted 1:10 with assay diluent. A volume of 100 μL of the diluted samples was added per well to precoated plates (SOD/MDA/IL-6/IFN-γ/TNF-α/IL-1β). The plates were at 37°C for 1 h, followed by washing three times with 300 μL wash buffer. Liquid was completely decanted, and plates were tapped dry on absorbent paper. A volume of 100 μL HRP-conjugated detection antibody was added per well. Plates were protected from light and incubated at 37°C for 1 h. After the final wash, 100 μL TMB substrate was added and developed in the dark at room temperature for 30 min. The reaction was stopped with 50 μL of 2M H₂SO₄. Absorbance was measured at 450 nm (reference 570 nm) within 15 min. For data analysis, samples were considered positive if the OD value was ≥ 2.1 × the negative control mean.

### Western-blot analysis

Total protein was extracted from tissues using Minute™ Total Protein Extraction Kit (Invent Biotechnologies, USA) according to the manufacturer’s protocol. Protein samples (30 µg per lane) were separated by 10% SDS-PAGE at 80 V for 30 min followed by 120 V for 1 h. Proteins were transferred to NC membranes (0.45 μm, Millipore) at 100 V for 90 min at 4 °C through Wet Transfer Method. After blocking with 5% skim milk at room temperature for 2 h, the primary antibodies (Anti-Jak1/2, Anti-p-Jak1/2, Anti-Stat1/3, Anti-p-Stat1/3) was diluted at 1:1000 and incubated overnight at 4 °C. The secondary antibody HRP-conjugated goat anti-rabbit IgG was diluted at 1:5000 and incubated 1.5 h at room temperature. NC membranes (Amersham Protran, Germany) were developed using Enhanced Chemiluminescence Reagent (Thermo Fisher Scientific, USA). Images were captured using ChemiDoc MP Imaging System (Bio-Rad, USA). Densitometric analysis performed using the Image J v1.53t (USA), band intensities were quantified using the Image J v1.53t with local background subtraction. Target protein expression levels were normalized to GAPDH^1^.

### Immunofluorescence staining

The sections were fixed, and subjected to antigen retrieval, membrane permeabilization, and blocking. They were then incubated at 4 °C overnight with CD68 antibody (green) for macrophages, CD11b antibody (red) for M1 macrophages, IL-17 antibody (red) for Th17 cells, and FoxP3 antibody (green) for Treg cells. the sections were stained with the respective antibodies and DAPI for nuclear staining. Then, the sections were mounted, and images were taken. The positive area of immunofluorescence was calculated using the Image J v1.53t^22^.

### 16 S rRNA analysis

The cecal and gastric contents were collected from the control, model, and H-sipeimine groups and stored at − 80℃ after being snap frozen in liquid nitrogen. All samples were detected by Beijing BIOMARKER TECHNOLOGIES Co., Ltd (Beijing, China). Community richness and diversity of the gut microbiota in each group were confirmed by microbial DNA extraction, 16 S rRNA gene amplicon sequencing, sequence analysis, bioinformatics and statistical analysis.

### Statistical analysis

Statistical tests were carried out using GraphPad Prism v 7.0. Multiple comparisons were conducted with one-way analysis of variance followed by Dunnett’s multiple comparison test. Statistical significance was defined as a p-value < 0.05. All values are presented as the mean ± standard deviation.

## Results

### Sipeimine ameliorates ethanol-induced gastric ulcer symptoms

The symptoms of gastric ulcer in mice (low body weight, large gastric injury area, high gastric injury index, and elevated histological scores) were significantly alleviated with sipeimine treatment. The body weight changes of mice model were significantly higher (*p* < 0.05) than those of sipeimine mice after 7 days (Fig. 1C). The area of gastric injury and the gastric injury index of the model group were markedly higher (*p* < 0.05) than those of the sipeimine group (Fig. 1D-F).

**Fig. 2:**
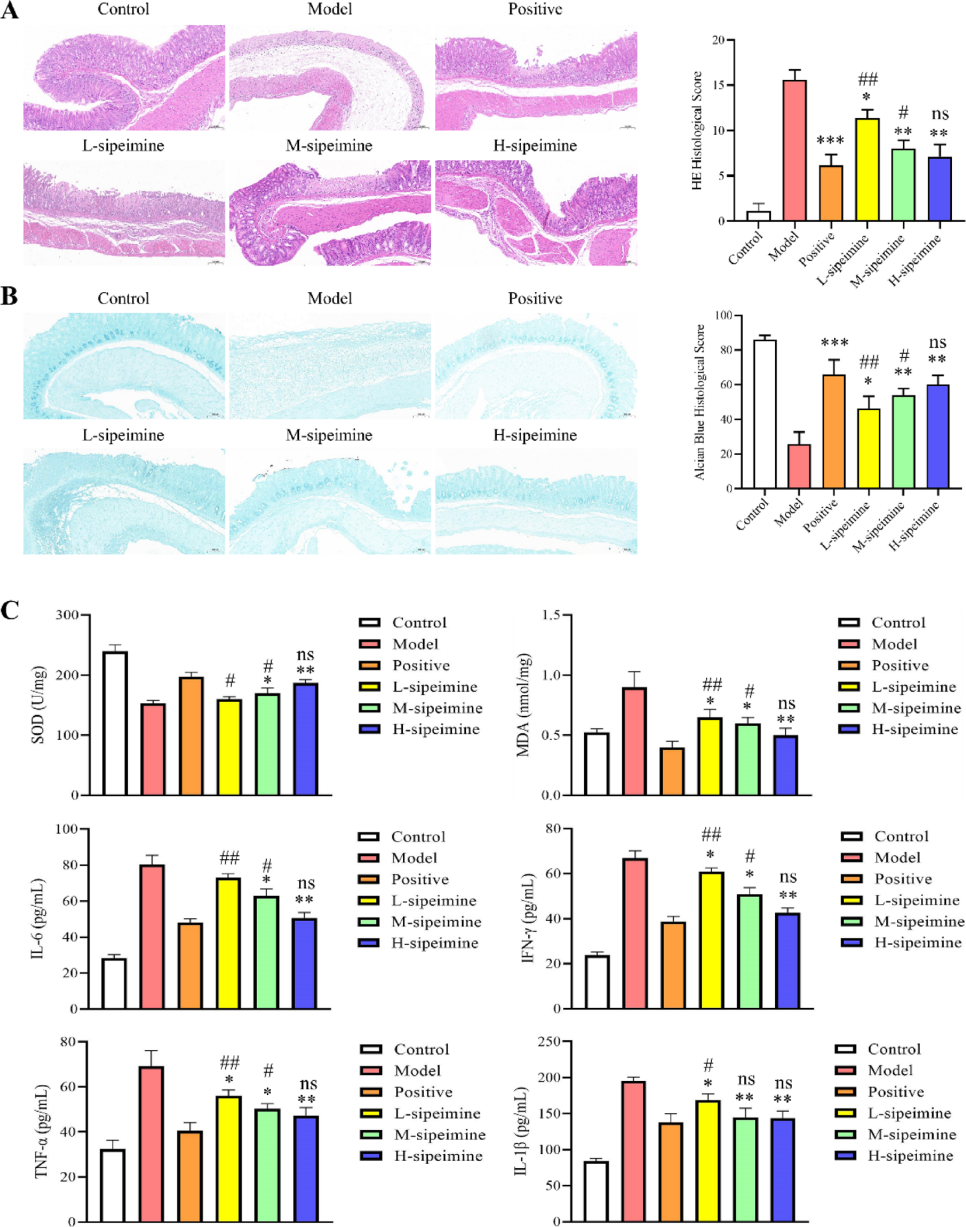
Effects of sipeimine on the histopathological scores and inflammatory cytokines levels of gastric ulcer mice (100× magnification). (**A**) HE images and histological scores. (**B**) AB images and histological scores. (**C**) Levels of SOD, MDA, IL-6, IFN-γ, TNF-α, and IL-1β in serum. Data are presented as the mean ± SD (*p < 0.05, **p < 0.01, ***p < 0.001 compared to the model group; ^#^p< 0.05, ^##^p < 0.01 compared to the positive group; ns, not significant).

Results from the HE staining and histological scores showed clear epithelial cell death, edema, and severe bleeding in the model group (Fig. 2A). Meanwhile, the AB staining and histological scores also showed that the mucosal integrity and acid mucus content were remarkably higher (*p* < 0.01) in the sipeimine groups (2, 4, and 8 mg/kg) than in the model group (Fig. 2B).

### Sipeimine reduces inflammatory cytokines in gastric ulcer mice

SOD, MDA, IL-6, IFN-γ, TNF-α and IL-1β levels in the sipeimine groups (2, 4 and 8 mg/kg) were noticeably lower (*p* < 0.05) than in the model group (Fig. 2C). These results indicated that sipeimine was able to remarkably suppress the inflammatory stress in the ethanol-induced gastric ulcer model in mice.

### Sipeimine inhibits jak-stat pathway activation

**Fig. 3:**
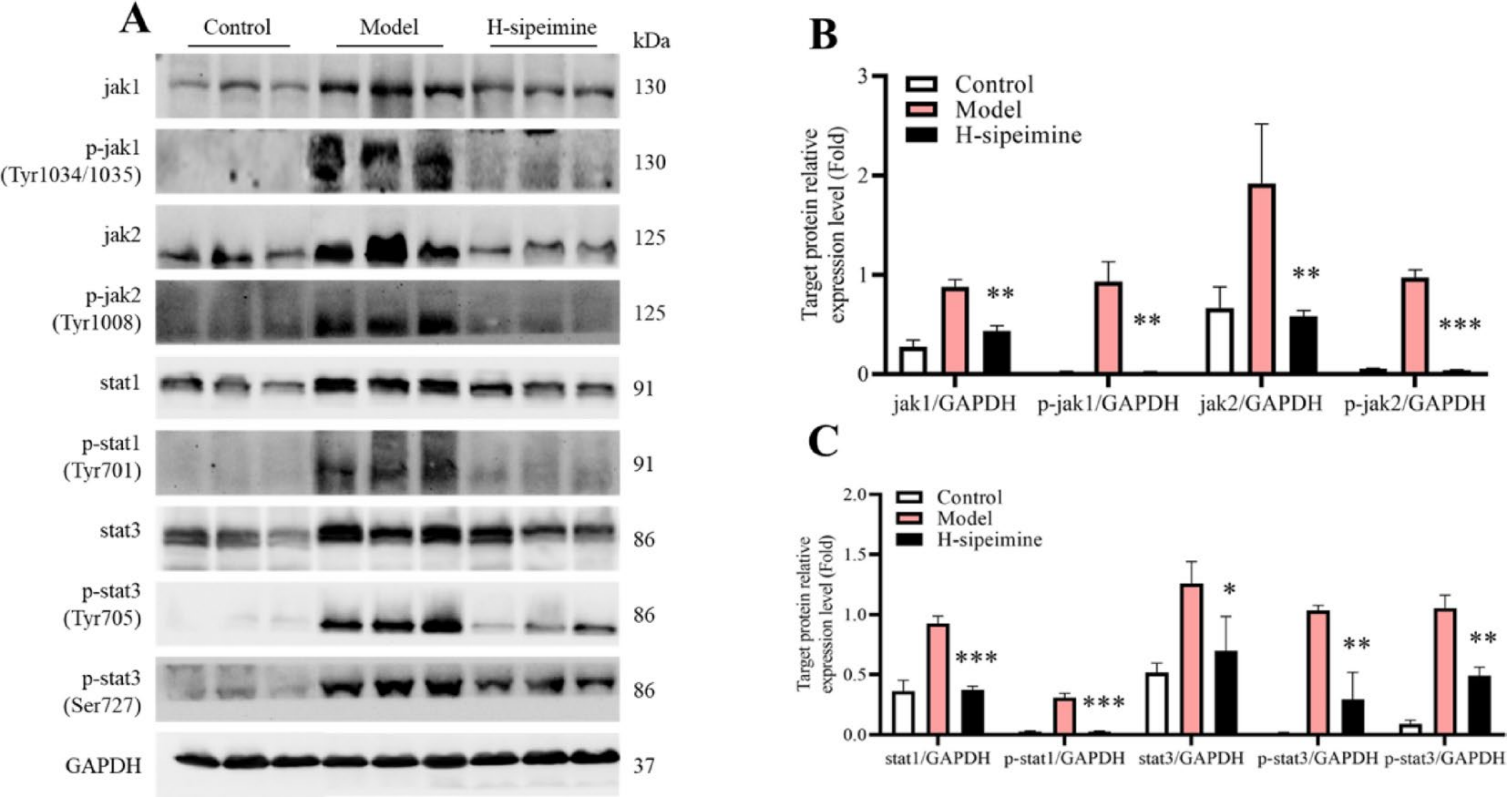
Effects of sipeimine on the Jak-Stat pathway. Effects of sipeimine on the Jak-Stat pathway. (**A**), (**B**) and (**C**) Western-blot results of Jak-Stat pathway in the mouse stomach. Data are presented as the mean ± SD (*p < 0.05, **p < 0.01, ***p < 0.001, compared to the model group).

Since high-dose sipeimine had a better improvement in ethanol-induced gastric ulcers, the H-sipeimine group was selected to evaluate the mechanism of action of sipeimine against gastric ulcers. Western blot results are presented in Fig. 3. Compared to the H-sipeimine group, the model group showed remarkably upregulated expression of Jak1/2, Stat1/3, p-Jak1/2, p-Stat1/3 in the gastric tissue (all *p* < 0.05).

### Sipeimine suppresses macrophage m1 polarization

**Fig. 4:**
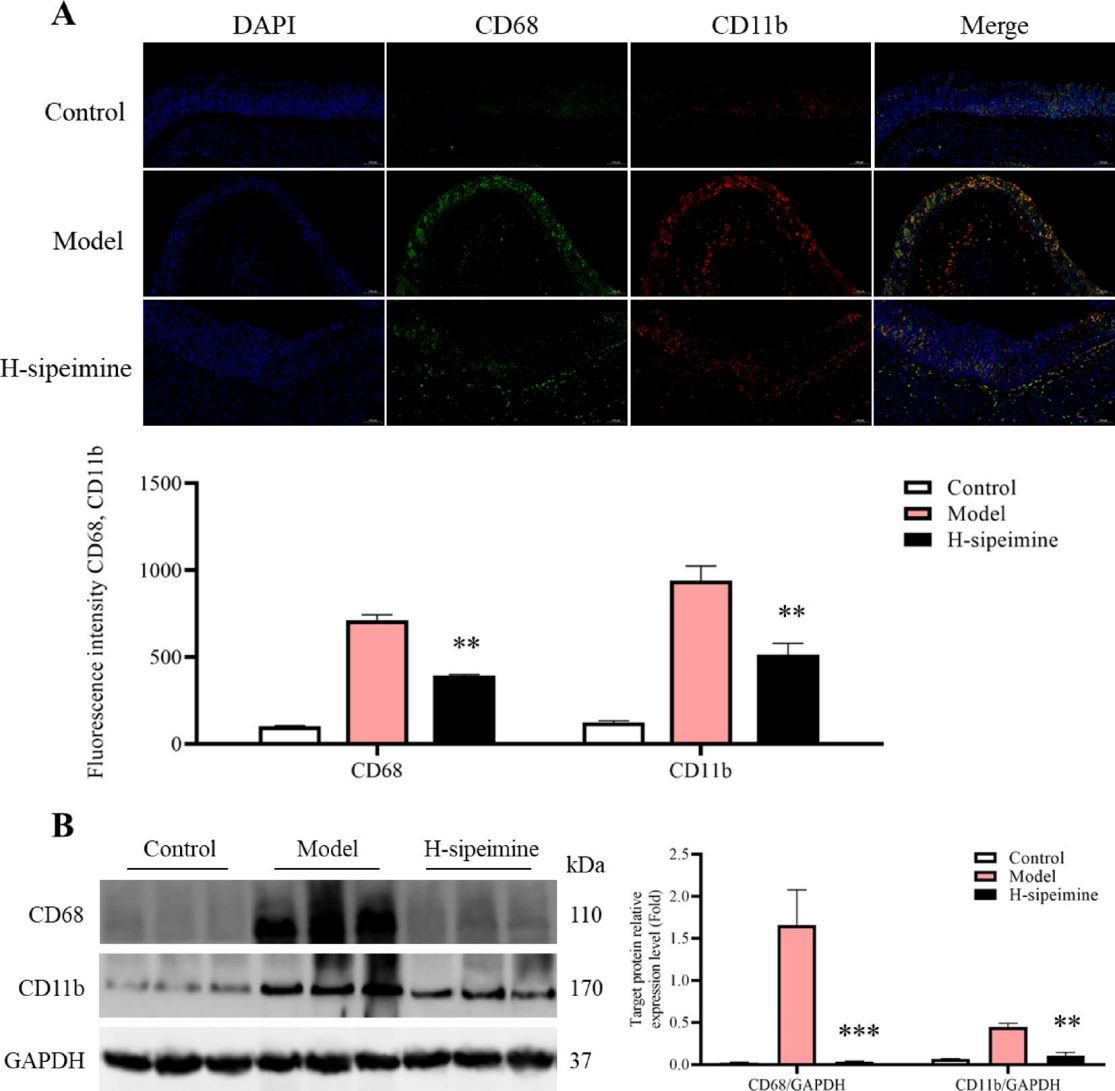
Effects of sipeimine on the macrophage M1 polarization (200×). (**A**) Immunofluorescence assessment and fluorescence intensity. (**B**) Western blot analysis. Data are presented as the mean ± SD (**p < 0.01, ***p< 0.001, compared to the model group).

To study the influence of sipeimine on M1 polarising macrophages in mice. Immunofluorescence and western blotting data revealed that CD68 and CD11b levels were significantly decreased (*p* < 0.01) in the H-sipeimine group compared to the model group (Fig. 4A-B). These results demonstrated that sipeimine might inhibited macrophage M1 polarization.

### Sipeimine adjusts the balance of th17/treg cells

**Fig. 5:**
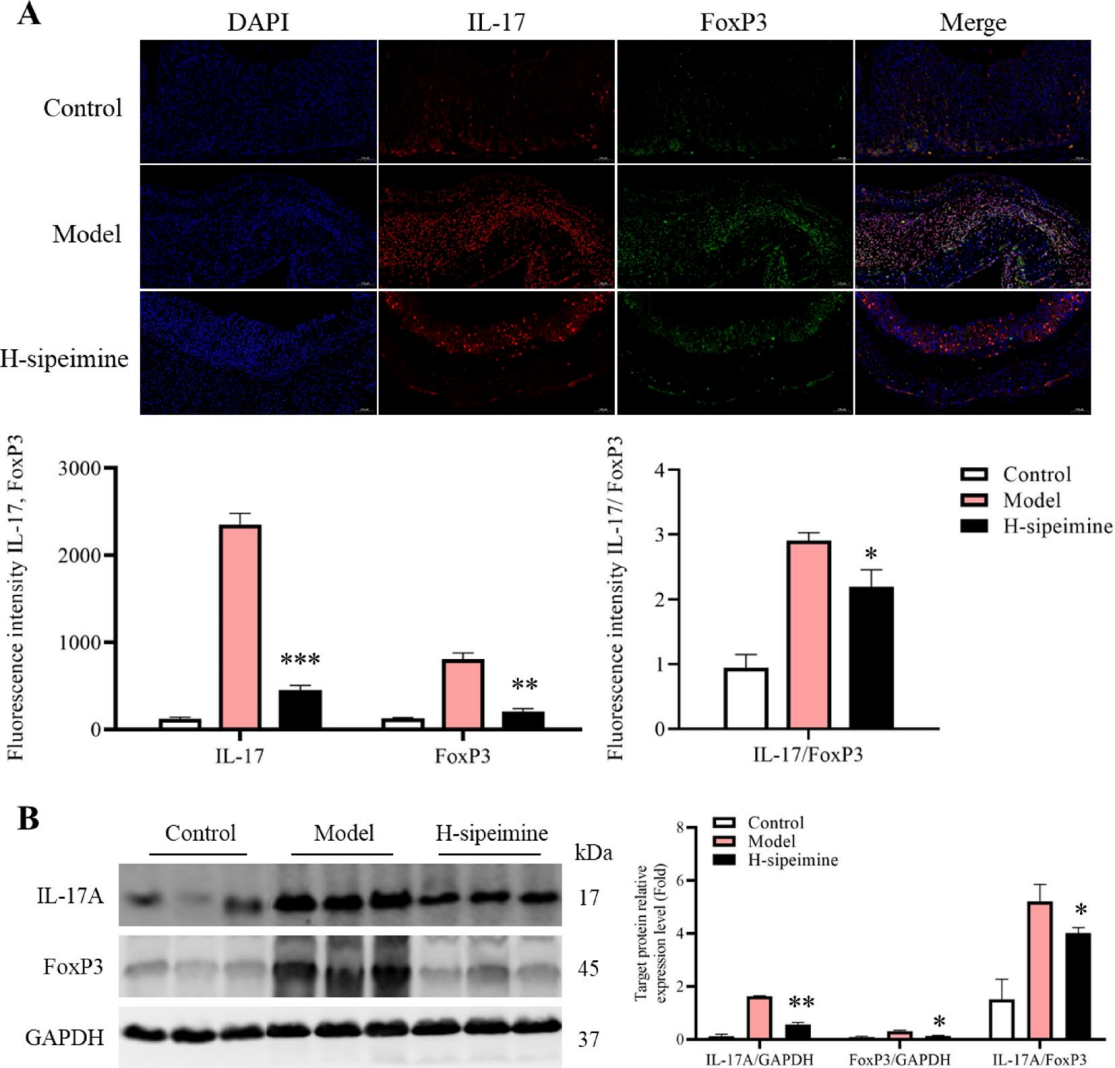
Effects of sipeimine on the Th17/Treg cells balance (200×). (**A**) Immunofluorescence assessment and fluorescence intensity. (**B**) Western blot analysis. Mean ± SD (*p < 0.01, **p< 0.01, ***p < 0.001, compared to the model group).

To determine the effects of sipeimine on the balance of Th17/Treg cells, we performed immunofluorescence and western blotting. These data showed that the IL-17, FoxP3, and IL-17/FoxP3 levels in the sipeimine group were significantly lower (*p* < 0.05) than those in the model group (Fig. 5A-B). These data indicated that sipeimine might adjust the Th17/Treg cells balance.

### Sipeimine improves gut-gastro microbiota in a gastric ulcer mouse model

**Fig. 6:**
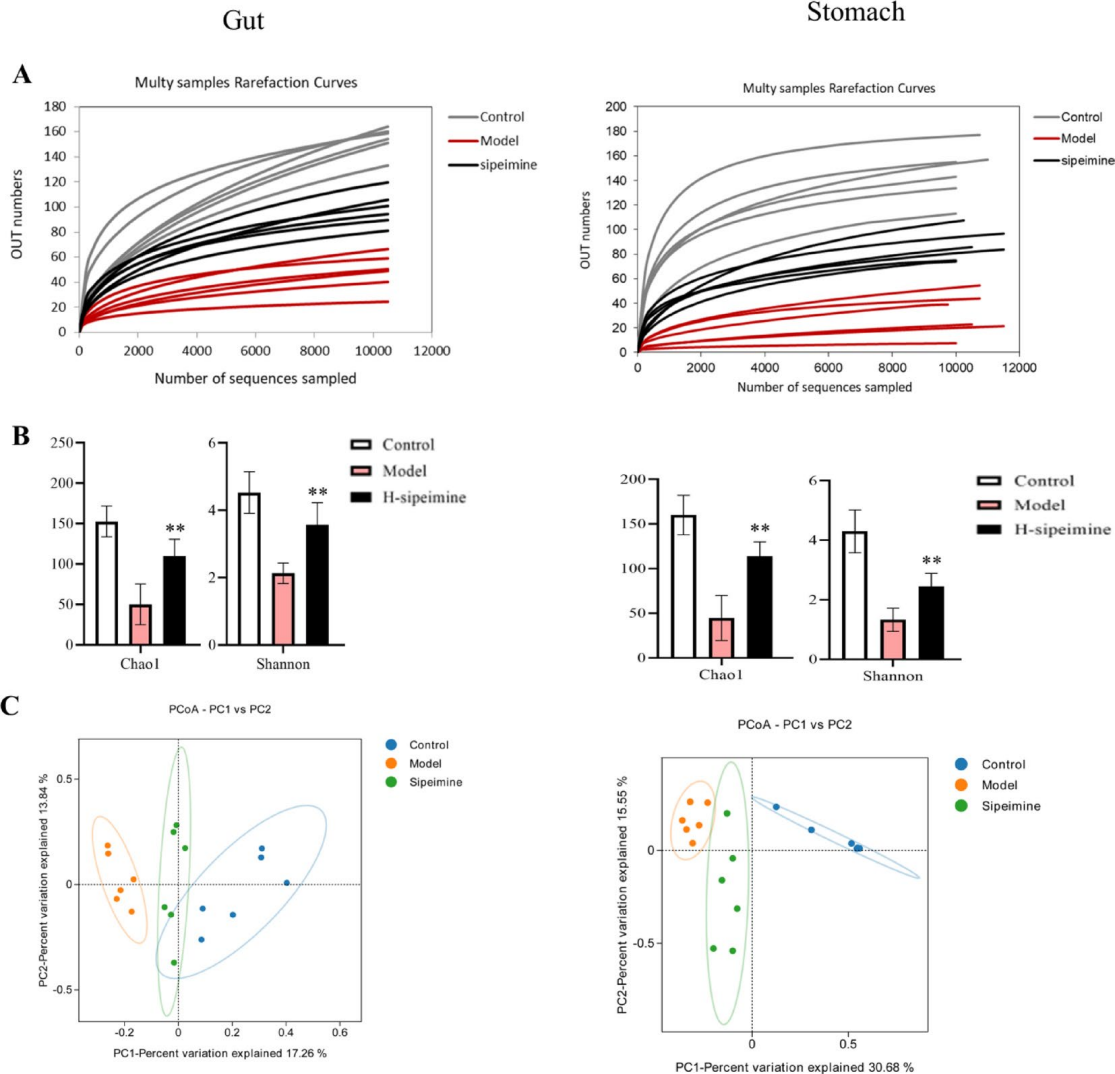
Effects of sipeimine on the gut and gastro microbiome structure. (**A**) Rarefaction curve. (**B**) Community richness (Chao1) and community diversity (Shannon). (**C**) PCoA analysis.

The bacterial diversity shifts in the cecum and gastrocnemius tissues were detected by using the 16 S rRNA analysis. Each sample’s Sobs index rarefaction curve fully reflected diversity in all samples (Fig. 6A). The community richness and community diversity were significantly enhanced in the H-sipeimine group compared to the model group (*p* < 0.05; Fig. 6B). These data indicated that the diversity of the gut-gastro microbiota of gastric ulcer mice could be improved by sipeimine treatment. Principal Coordinate Analysis (PCoA) data showed that the aberrant structure of gut-gastro microbiota could be significantly ameliorated with H-sipeimine in an ethanol-induced gastric ulcer mouse model (Fig. 6C).

**Fig. 7:**
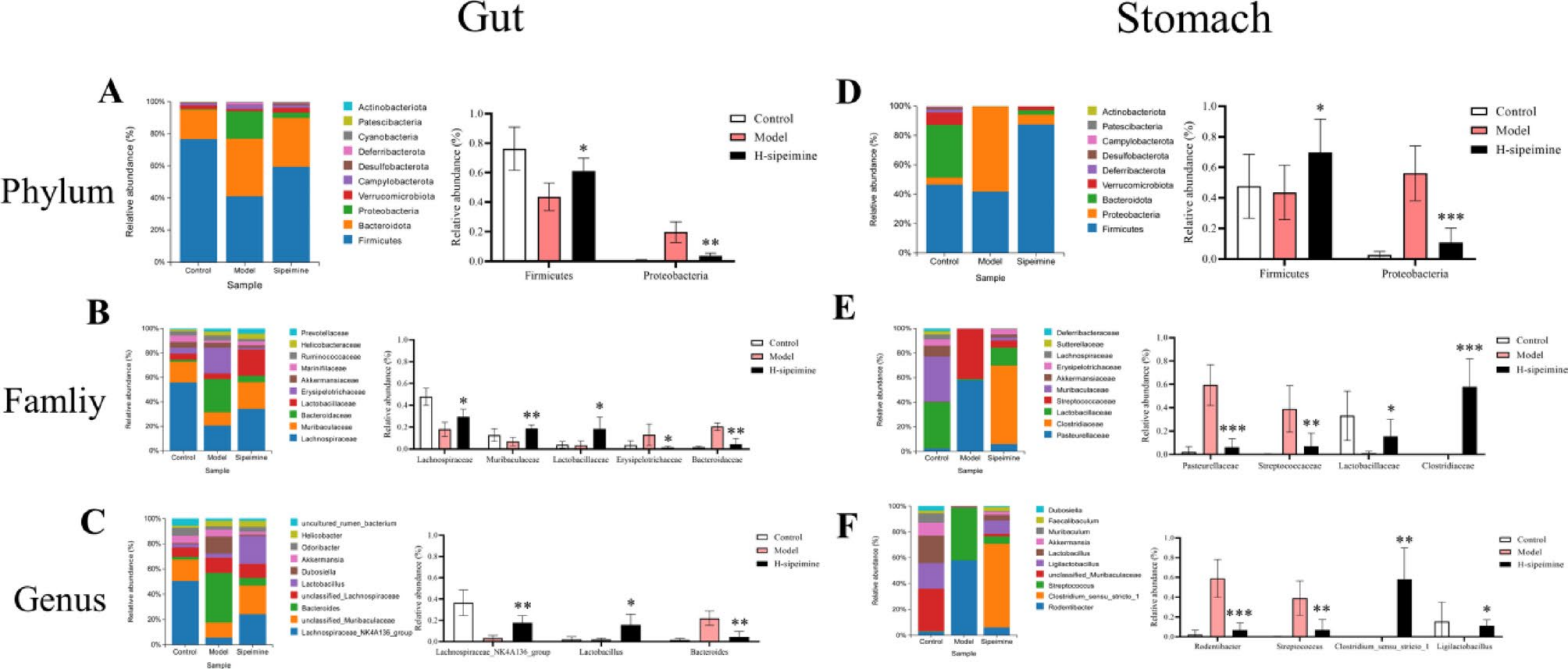
Effects of sipeimine on the gut and gastro microbiome diversity and composition. (**A**) and (**D**) The phylum level. (**B**) and (**E**) The family level. (**C**) and (**F**) The genus level. Data are presented as the mean ± SD (*p < 0.05, **p < 0.01, ***p < 0.001, compared to the model group).

**Fig. 8:**
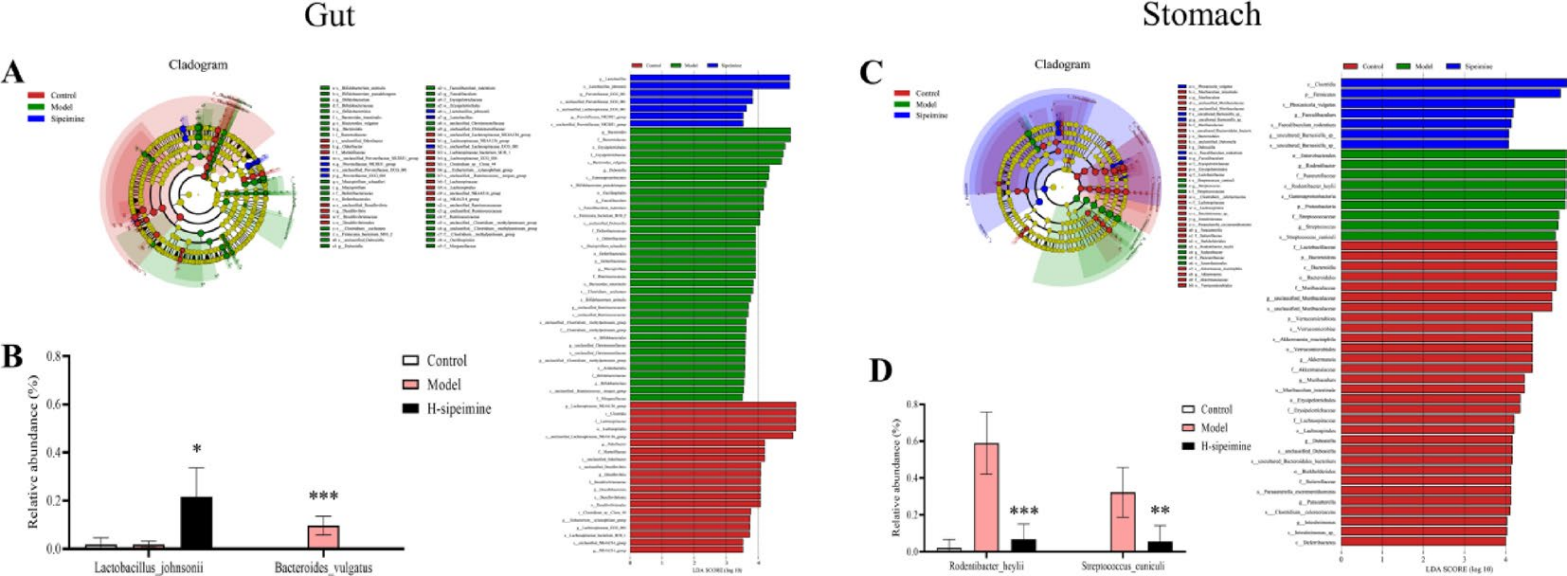
Effects of sipeimine on the species level.(**A**) and (**C**) LEfSe analysis. (**B**) Relative abundance of *Lactobacillus_johnsonii* and* Bacteroides_vulgatus *in gut microbiota. (**D**) Relative abundance of *Rodentibacter_heylii *and* Streptococcus_cuniculi *in gastro microbiota. Data are presented as the mean ± SD (*p < 0.05, **p < 0.01, ***p < 0.001, compared to the model group).

As shown in Figs. 7 and 8 and H-sipeimine affects bacterial enrichment at different levels.

At the phylum level, compared to the H-sipeimine group, the model group significantly reduced the relative abundance of *Firmcutes* while remarkably increased that of *Proteobacteria* in the gut microbiota (Fig. 7A). At the family level, for gut microbiota, *Lachnospiraceae*,* Muribaculaceae*, and *Lactobacillaceae* were less abundant, whereas *Erysipelotrichaceae* and *Bacteroldaceae* were more abundant in the model group than in the H-sipeimine group (*p* < 0.05; Fig. 7B). At the genus level, for gut microbiota, the abundances of *Lachnospiraceae_NK4A136_group* and *Lactobacillus* were remarkably higher (*p* < 0.05) in the H-sipeimine group than in the model group, whereas the relative abundances of *Bacteroides* were remarkably lower (*p* < 0.01) in the H-sipeimine group than in the model group (Fig. 7C). At the phylum level, compared to the H-sipeimine group, the model group significantly reduced the relative abundance of *Firmcutes* while remarkably increased that of *Proteobacteria* in the gastro microbiota (Fig. 7D). At the family level, for gastro microbiota, *Pasteurellaceae* and *Streptococcaceae* were less abundant, whereas *Lactobacillaceae* and *Clostridiaceae* were more abundant in the H-sipeimine group than in the model group (*p* < 0.05; Fig. 7E). At the genus level, for gastro microbiota, the abundances of *Rodentibacter* and *Streptococcus* were remarkably lower (*p* < 0.01) in the H-sipeimine group than in the model group, whereas the relative abundances of *Clostridium sensu stricto l* and *Ligilactobacillus* were remarkably higher (*p* < 0.05) in the H-sipeimine group than in the model group (Fig. 7F).

LEfSe analysis was used to identify the effect of H-sipeimine treatment on gut gastro microbiota diversity in ethanol-induced gastric ulcer mice (Fig. 8A, C). At the species level, for gut microbiota, compared to the H-sipeimine group, the model group demonstrated markedly downregulated relative abundances of *Lactobacillus_johnsonii* (*p* < 0.05), but markedly upregulated relative abundances of *Bacteroides_vulgatus* (*p* < 0.001) (Fig. 8B). For gastro microbiota, compared to the model group, the H-sipeimine group markedly downregulated the relative abundances of *Rodentibacter_heylii* and *Streptococcus_cuniculi* (*p* < 0.01; Fig. 8D). In conclusion, these data indicated that sipeimine has a beneficial effect on improving gut-gastro microbiota dysbiosis in a gastric ulcer mouse model.

## Discussion

Gastric ulcers are a major pervasive gastrointestinal disease characterized by the secretion of inflammatory factors, neutrophil infiltration and oxidative stress^23^. Macrophage polarization and Th17/Treg cells balance contribute to the maintenance of gastrointestinal homeostasis and play an important role in the pathogenesis of gastrointestinal inflammation, similar to gastric ulcer^3,6^ and ulcerative colitis^24,25^. Macrophages are commonly found in two distinct sub-populations(the pro-inflammatory M1 macrophage and the anti-inflammatory M2 macrophage)^26,27^. Pro-inflammatory cytokines such as TNF-α and IL-17 are produced by pro-inflammatory Th17 cells^28^. Treg cells provide anti-inflammatory benefits and produce anti-inflammatory cytokines like TGF-β and IL-10^29^. Therefore, in the inflammatory response, it is particularly essential to maintain the Th17/Treg cells balance^30^.

Jak-Stat is a classic signaling pathway that is essential for the regulation of Th17/Treg cells balance^31,32^and monocyte differentiation^21^. Some studies have shown that Stat1 contributes to modulate macrophage M1 polarization^33^. Stat3 participates in Th17 differentiation and negatively regulates Treg formation^34^. In this research, control to model group, H-sipeimine group demonstrated dramatically reduced expression of Jak1/2, Stat1/3, p-Jak1/2, and p-Stat1/3 (*p* < 0.05). The M1 macrophages and Th17 cells levels were markedly lower in the H-sipeimine group than in the model group. These results indicated that sipeimine might suppress the M1 macrophage and Th17 cells activities through inhibiting the Jak-Stat pathway.

Long-term excessive alcohol consumption could alter the diversity of the gastrointestinal microbiota and influence microbial metabolic pathways. The related metabolites, especially short chain fatty acids, could alleviate alcohol-induced gastric injury by maintaining a stable gastrointestinal environment, relieving gut inflammation, and recovering mucosal barrier integrity^35,36,37,38^. Due to the presence of gastric acid, the stomach is considered to be an organ with few bacteria. The results of 16 S rRNA analysis has led to the discovery of an increased proportion of bacterial species. In this investigation, we constructed an ethanol-induced gastric ulcer mouse model using multiple intragastrical administration of ethanol. This method could affect the stability and resilience of the gut-gastro microbiome and effectively change the structure of the gut-gastro microbiota in mice model. For gut microbiota, compared with the model group, the abundance of *Lactobacillus_johnsonii* in the H-sipeimine group increased by 11.65-fold, while the abundance of *Bacteroides_vulgatus* decreased by 174.97-fold. *Lactobacillus_johnsonii* is a potential probiotic candidate with an antagonistic pathogen that modulates mucosal and systemic immune responses, reduces chronic inflammation, regulates metabolic disorders, and enhances the epithelial barrier^39^. *Bacteroides_vulgatus* is recognized as a pathogenic bacteria^40^. For gastro microbiota, compared to the model group, the abundances of *Rodentibacter_heylii* and *Streptococcus_cuniculi* in the H-sipeimine group decreased by 6.03-fold and 8.83-fold, respectively. *Rodentibacter_heylii* is recognized as an opportunistic pathogenic bacterium^41^. *Streptococcus_cuniculi* is an opportunistic bacterium that can cause corneal lesions in mice^42^. These data indicated that sipeimine inhibited the levels of M1 macrophages and Th17 cells by regulating Jak-Stat pathway activation. Sipeimine also improved gut-gastro microbiota dysbiosis.

## Conclusion

Both excessive activation of the Jak-Stat pathway and gut-gastro microbiota dysbiosis intensified the inflammatory injury of alcohol-induced gastric ulcers. In this research, sipeimine inhibited the Jak-Stat pathway and affected pro-inflammatory cell levels. Sipeimine also regulates the composition of gut microbiota by increasing the abundance of *Lactobacillus_johnsonii* and decreasing the abundance of *Bacteroides_vulgatus*, *Rodentibacter_heylii* and *Streptococcus_cuniculi*. Sipeimine inhibits the secretion of pro-inflammatory cytokine and mitigates the dysbiosis of gut-gastro microbiota to improve ethanol-induced gastric ulcer symptoms.

## Electronic supplementary material

Below is the link to the electronic supplementary material.

Supplementary Material 1

Supplementary Material 2

## Data Availability

Data will be made available on request to corresponding author.

## Abbreviations

ACE: Angiotensin converting enzyme
APC: Allophycocyanin
CST: Cell Signal Technology
DAPI: 4`,6-Diamidino-2-Phenylindole
Dex: Dexamethasone
ECL: Electronics Components Laboratory Chemiluminescence Solution
E. coli: Escherichia coli
FITC: Fluorescein Isothiocyanate
FoxP3: Forkhead box P3
HE: Hematoxylin and eosin
JAK1/2: Janus kinases 1/2
HRP: Horseradish Peroxidase
IL-6: Interleukin-6
IL-1β: Interleukin 1 bate protein
INF-γ: Interferon-γ
IL-17A: Interleukin 17 Receptor A
mAb: Monoclonal Ab
MCE: Med Chem Express
MDA: Malondialdehyde
SOD: Superoxide dismutase
SDS-PAGE: Sodium Dodecyl Sulfate Polyacrylamide Gel Electrophoresis
STAT1: Signal Transducer and Activator of Transcription 1
Treg: T regulatory
Th: T helper
TNF-α: Tumor Necrosis Factor α
PE: Phycoerythrin
p-STAT1: Phosphorylation Signal Transducer and Activator of Transcription 1
PVDF: Polyvinylidene Fluoride
PCoA: Principal co-ordinates Analysis
