# Supplementary material for: Sipeimine reduces ethanol-induced gastric ulcer in mice by suppressing Jak-Stat activation and restoring gut microbiota balance
Source: Sci Rep. 2025 Aug 6;15:28683. doi: 10.1038/s41598-025-12050-2 (PMC12329034; doi:10.1038/s41598-025-12050-2)
Supplement: Supplementary file 1 — Supplementary Material 1 [file 41598_2025_12050_MOESM1_ESM.pptx]

## Slide 1
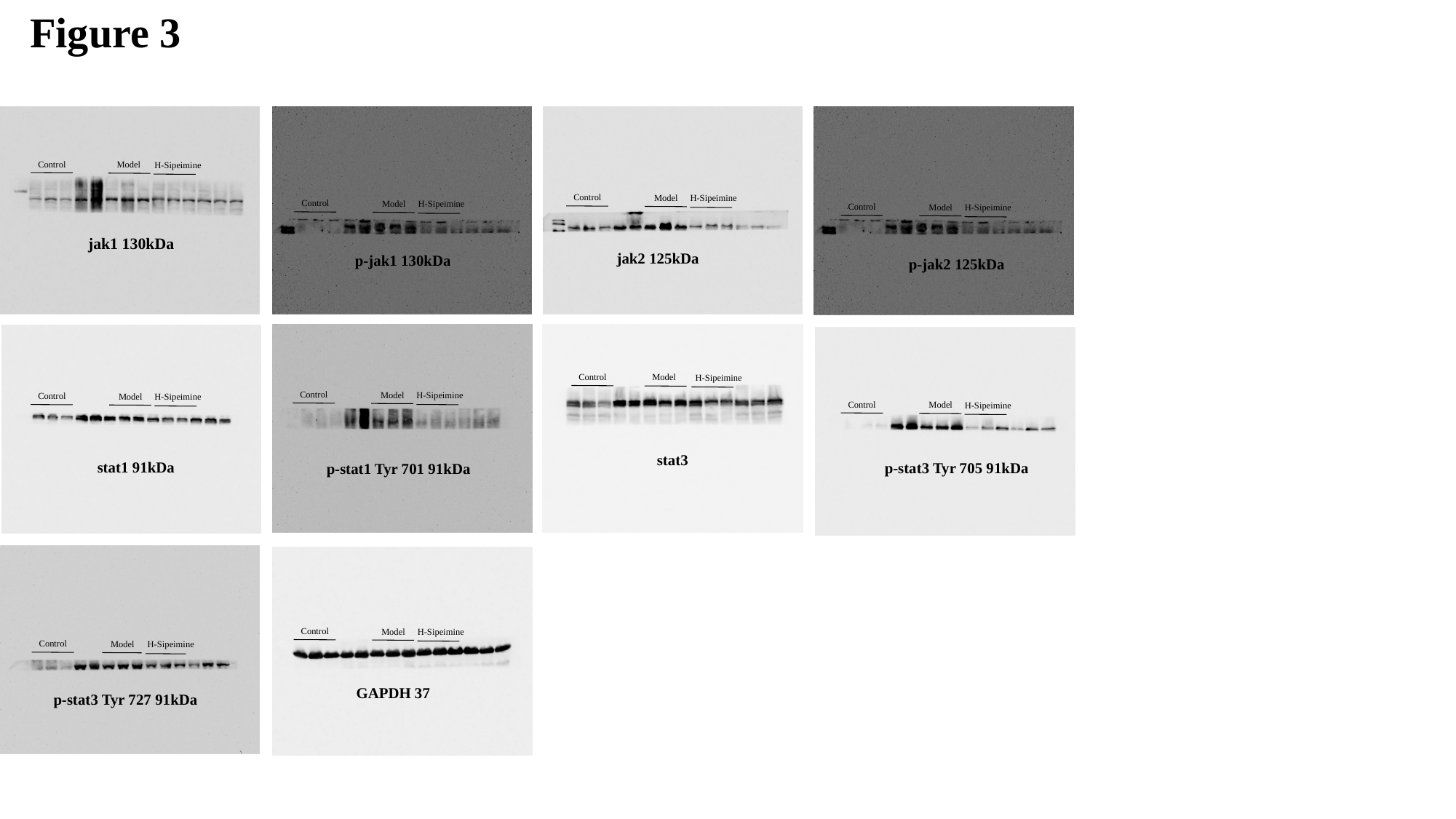

Figure 3
p-jak1 130kDa
p-jak2 125kDa
jak2 125kDa
Control
Model
H-Sipeimine
Control
Model
H-Sipeimine
Control
Model
H-Sipeimine
Control
Model
H-Sipeimine
jak1 130kDa
Control
Model
H-Sipeimine
Control
Model
H-Sipeimine
Control
Model
H-Sipeimine
Control
Model
H-Sipeimine
stat3
stat1 91kDa
p-stat3 Tyr 705 91kDa
p-stat1 Tyr 701 91kDa
Control
Model
H-Sipeimine
Control
Model
H-Sipeimine
GAPDH 37
p-stat3 Tyr 727 91kDa

## Slide 2
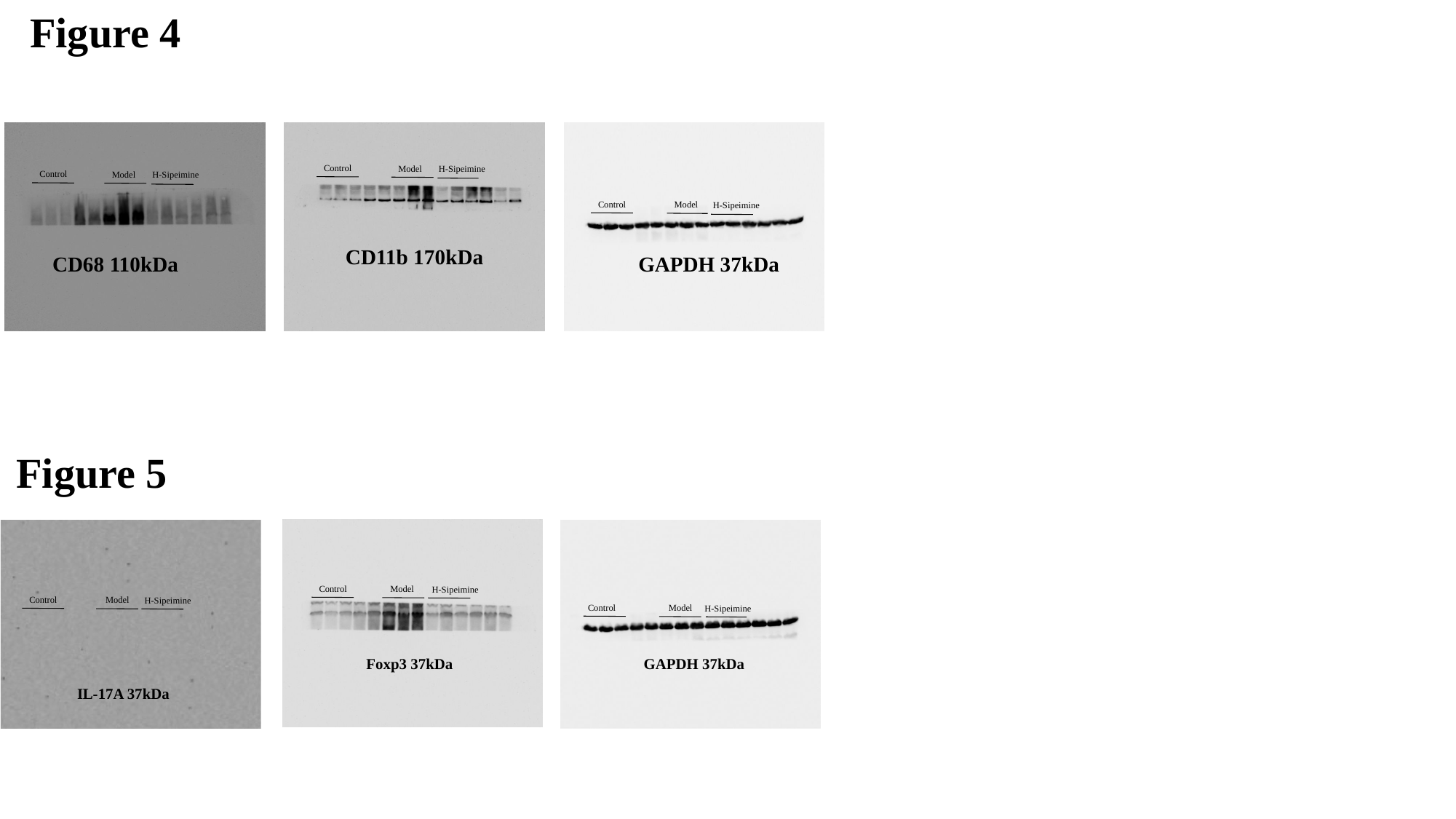

Figure 4
Control
Model
H-Sipeimine
Control
Model
H-Sipeimine
Control
Model
H-Sipeimine
CD11b 170kDa
GAPDH 37kDa
CD68 110kDa
Figure 5
Control
Model
H-Sipeimine
Control
Model
H-Sipeimine
Control
Model
H-Sipeimine
Foxp3 37kDa
GAPDH 37kDa
IL-17A 37kDa
