# Supplementary material for: Sipeimine reduces ethanol-induced gastric ulcer in mice by suppressing Jak-Stat activation and restoring gut microbiota balance
Source: Sci Rep. 2025 Aug 6;15:28683. doi: 10.1038/s41598-025-12050-2 (PMC12329034; doi:10.1038/s41598-025-12050-2)

Author Agreement

Dear Editor,

We the undersigned declare that this manuscript entitled "***Sipeimine alleviates ethanol-induced gastric ulcer by inhibiting Jak-Stat activation and mitigating gut-gastro microbiota dysbiosis in mice***" is original, has not been published before and is not currently being considered for publication elsewhere.

We confirm that the manuscript has been read and approved by all named authors and that there are no other persons who satisfied the criteria for authorship but are not listed. We further confirm that the order of authors listed in the manuscript has been approved by all of us.

We understand that the Corresponding Author is the sole contact for the Editorial process. She is responsible for communicating with the other authors about progress, submissions of revisions and final approval of proofs.

All data were generated in-house, and no paper mill was used. All authors agree to be accountable for all aspects of work ensuring integrity and accuracy.

CRediT author statement

Xia Yang Writing - original draft and Formal analysis. **Yue Li**: Writing - original draft and Formal analysis. **Bing Bai**: Writing - original draft and Formal analysis. **Qinlei Fan**: Conceptualization. **Fei Liu**: Project administration. **Shimin Xie**: Conceptualization. **Yaru Li**: Visualization. **Xiao Li**: Supervision. **Jicheng Han**: Writing - review & editing. **Yiquan Li**: Writing - review & editing. **Guangze Zhu**: Writing - review & editing.

Yilong Zhu: Writing - review & editing. **Ningyi Jin**: Supervision. All the listed authors have read and approved the submitted manuscript.

Signed by all authors as follows:

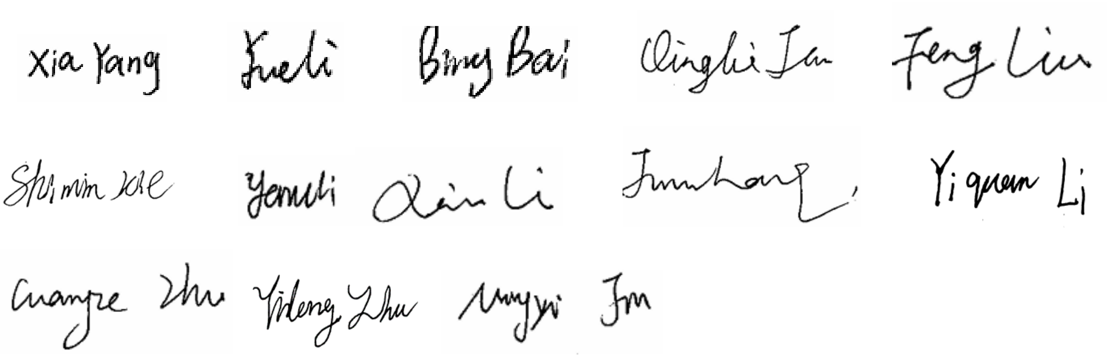

Supplement: Supplementary file 2 — Supplementary Material 2 [file 41598_2025_12050_MOESM2_ESM.pdf]
